# 3D graphene preparation via covalent amide functionalization for efficient metal-free electrocatalysis in oxygen reduction

**DOI:** 10.1038/srep43279

**Published:** 2017-02-27

**Authors:** Mohammad Shamsuddin Ahmed, Young-Bae Kim

**Affiliations:** 1 Department of Mechanical Engineering, Chonnam National University, Gwangju, Republic of Korea

## Abstract

3D and porous reduced graphene oxide (rGO) catalysts have been prepared with sp^3^-hybridized 1,4-diaminobutane (sp^3^-DABu, rGO-sp^3^-rGO) and sp^2^-hybridized 1,4-diaminobenzene (sp^2^-DABe, rGO-sp^2^-rGO) through a covalent amidation and have employed as a metal-free electrocatalyst for oxygen reduction reaction (ORR) in alkaline media. Both compounds have used as a junction between functionalized rGO layers to improve electrical conductivity and impart electrocatalytic activity to the ORR resulting from the interlayer charge transfer. The successful amidation and the subsequent reduction in the process of catalyst preparation have confirmed by X-ray photoelectron spectroscopy. A hierarchical porous structure is also confirmed by surface morphological analysis. Specific surface area and thermal stability have increased after successful the amidation by sp^3^-DABu. The investigated ORR mechanism reveals that both functionalized rGO is better ORR active than nonfunctionalized rGO due to pyridinic-like N content and rGO-sp^3^-rGO is better ORR active than rGO-sp^2^-rGO due to higher pyridinic-like N content and π-electron interaction-free interlayer charge transfer. Thus, the rGO-sp^3^-rGO has proven as an efficient metal-free electrocatalyst with better electrocatalytic activity, stability, and tolerance to the crossover effect than the commercially available Pt/C for ORR.

Graphene oxide (GO)^1^ is the oxidized and exfoliated sheet of sp^2^-hybridized graphene that carries various oxygenated groups, such as epoxide, hydroxyl, carbonyl, and carboxyl. Among all the oxygenated groups, the carboxyl group of GO is the most suitable site for reacting with an amine group to form an amide bond through condensation reaction^2^,^3^,^4^,^5^. However, graphene itself has zero band gap, a fact that poses a considerable barrier to its application in digital electronic devices, such as semiconductors, sensors, and electrocatalysts^2^. Several approaches, including implanting heteroatoms (e.g., N atom) onto a graphene plane, have been applied to overcome this disadvantage^2^,^6^,^7^. The N atom is considered as the best candidate among various heteroatoms (i.e., B, S, P, F and O) for carbon (C) substitution because of its five valence electrons that are capable of forming a strong interaction with adjacent C atoms.

In the reduced graphene oxide (rGO) lattice which prepared from GO by reduction reaction, N doping/functionalization creates a net positive charge in adjacent C atoms to facilitate oxygen reduction reaction (ORR) by changing the O_2_ chemisorption mode and by attracting electrons from the anode. This phenomenon has been proved through quantum mechanics calculations and subsequent experimental observations^8^,^9^,^10^. Tao *et al*.^11^ observed that the N atoms induced charge polarization of C atoms in graphitic plane could significantly improve ORR performance^10^,^12^,^13^. They also reported that the sp^3^-C-rich rGO could provide efficient catalysis to ORR because of their effective charge polarization^11^. In the meantime, Yang *et al*.^14^ also reported that their sp^3^-C-rich 3D N-graphene showed an excellent charge transport for ORR. Moreover, the sp^3^-C-rich carbon spheres showed the significantly improved ORR than sp^2^-C-rich carbon spheres which was reported by Sanetuntikul *et al*. earlier^15^. Recent studies have also confirmed that the N-induced charge transfer on a single rGO plane has a significant effect on the development of various graphene-based metal-free ORR catalysts for fuel cells (FCs)^9^,^11^,^16^. So far, the charge transfer between multi-layers of rGO (3D type) has not been investigated yet. Based on the above discussion, the interlayer charge transfer^17^ is predicted to be enhanced by the covalent functionalization of sp^3^-C-rich molecules in between rGO layers, thus facilitating ORR.

Huge effort has been expended in the search for sustainable and renewable sources of green and clean energy because of the increasing demand and environmental impact of traditional energy resources, such as, fossil fuels^18^,^19^. In this regard, FCs have received significant attention as next-generation energy sources^20^,^21^ because they directly generate electricity by electrochemically reducing oxygen and by oxidizing fuel into water as only the by-product. Direct ORR^8^,^21^,^22^ catalysis involving four electrons is an interesting research area because of its important role in the application of energy conversion devices, such as FCs and metal-air batteries in alkaline media^18^,^19^,^20^,^21^,^23^. Novel metals, such as platinum (Pt) and its alloy materials are regarded as the most efficient electrocatalysts for ORR in cathodes^24^,^25^. For example, ORR proceeds on through four electron transfer constantly for longer potential range at Pt-based electrodes which resultant from the quick-reaching at steady-state limiting diffusion current^26^,^27^. However, Pt-based materials are susceptible to the crossover effect caused by the diffusion of fuel molecules from the anode through the membrane to the cathode in FCs^9^, CO poisoning^10^,^28^, and poor durability^29^. Moreover, the cost reduction of FCs is greatly hampered by the high cost and limited supply of Pt usages^30^. Nevertheless, significant effort has been focused on the development of alternative catalysts, including nonprecious metal or metal-free carbon nanomaterial-based ORR catalysts, to overcome the aforementioned challenges^8^,^9^,^31^,^32^,^33^,^34^,^35^. Thus, efforts should be exerted to identify readily available and cost-effective alternative catalysts for cathodic ORR in FCs that also show comparable or even better catalytic effects than Pt in terms of quick-reaching at four electrons involved ORR while it is crucially important in order to increase the Faradic efficiency of FCs and to decrease the H_2_O_2_ production during ORR^22^.

In this study, we developed hierarchical porous rGO for using as a highly efficient metal-free ORR electrocatalyst through a covalent functionalization method via amidation reaction with sp^3^-hybridized 1,4-diaminobutane (sp^3^-DABu) (rGO-sp^3^-rGO) or sp^2^-hybridized 1,4-diaminobenzene (sp^2^-DABe) (rGO-sp^2^-rGO) as shown in Fig. 1. Both molecules containing amine groups in the terminal positions were used as the junction between rGO layers to impart electrocatalytic activity for the ORR resulting from the net positive charge caused by the N atoms of the adjacent C atoms. Particularly, the rGO-sp^3^-rGO showed better electrocatalytic activity, stability, and tolerance to the crossover effect than that of other tested samples including state-of-the-art Pt/C because of its interaction-free interlayer charge transfer.

## Results and Discussion

Figure 2a shows the TGA curves recorded under a nitrogen atmosphere for GO-COOH, rGO, rGO-sp^2^-rGO, and rGO-sp^3^-rGO samples. GO-COOH showed its poor thermal stability and considerable mass loss (16.4 wt %) at 100 °C were due to the removal of adsorbed water. As most of the weight was lost between 100 °C and 200 °C, the CO and CO_2_ released from the most labile functional groups and the total weight loss were approximately 99% at 600 °C, similar to that of GO and indicating similar natures under thermal condition^36^. By contrast, the dramatically improved thermal stability of nonfunctionalized rGO was observed; 6.9 and 59.9% of weight loss were found at 100 °C and 600 °C, respectively. These observations indicated low moisture content and a successful reduction of GO-COOH upon NaBH_4_ treatment. At 600 °C, rGO-sp^3^-rGO showed good thermal stability with approximately 38.8% weight loss, which was approximately 1.5 times lower than the thermal stability of rGO at 600 °C. Further weight loss of approximately 26% between 600 °C and 750 °C is similar to previous reports^37^. This result indicated the superior thermal stability of rGO-sp^3^-rGO that was most likely caused by its hierarchical porous structure resulting from the layer-by-layer connection through sp^3^-DABu^38^. A similar behavior was observed for rGO-sp^2^-rGO with a slightly low thermal stability (weight loss of 42.5% at 600 °C). Figure S4 compares the TGA of the three different rGO-sp^3^-rGO samples prepared with three different w/w ratios of GO–COOH and sp^3^-DABu.

**Figure 1:**
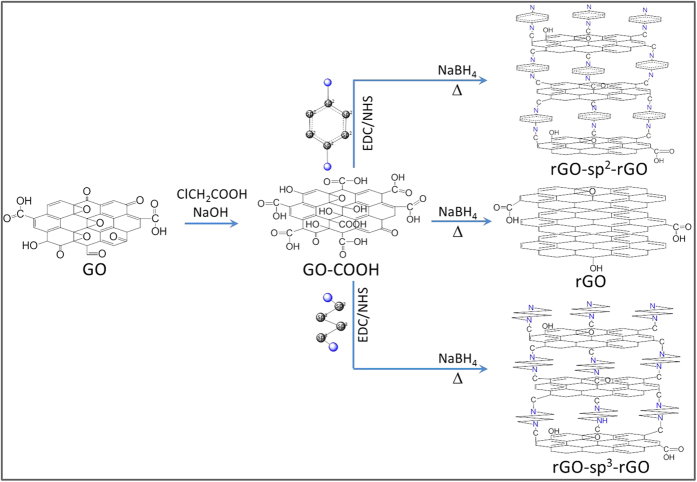
The schematic diagram of finished products synthesis (rGO, rGO-sp^2^-rGO and rGO-sp^3^-rGO).

XPS analysis elucidated the details of the chemical structure and bonding state changes during amidation with sp^3^-DABu or sp^2^-DABe molecule and GO reduction (as synthesized in Fig. 1). The conversion treatment was conducted with GO, and the C/O ratio of GO and GO–COOH was the same at *ca.* 2.1 (Figure S1a). In the C1s comparison, the GO–COOH sample showed a strong absorbance peak for O–C=O and a comparatively weak absorbance peak for C=O (Figure S1b). This observation indicated the successful conversion to the carboxyl group of the GO–COOH sheets. Figure S1c shows the XPS survey spectra for GO–COOH, rGO, rGO-sp^2^-rGO, and rGO-sp^3^-rGO. All the XPS survey spectra exhibited several distinct peaks at approximately 285.5 and 533.5 eV, which were attributed to C and O elements^39^,^40^. rGO-sp^2^-rGO and rGO-sp^3^-rGO XPS survey spectra showed a peak at approximately 400 eV for N along with C and O^8^,^12^,^41^. However, the C/O atomic ratio was significantly increased from 2.1 at *ca.* 5.9, 6.1, and 6.2 for rGO, rGO-sp^2^-rGO, and rGO-sp^3^-rGO samples, respectively. This result indicated the higher degree of oxygen-containing group reduction from the GO–COOH surface upon amidation and subsequent NaBH_4_ reduction^8^,^9^,^42^.

**Figure 2:**
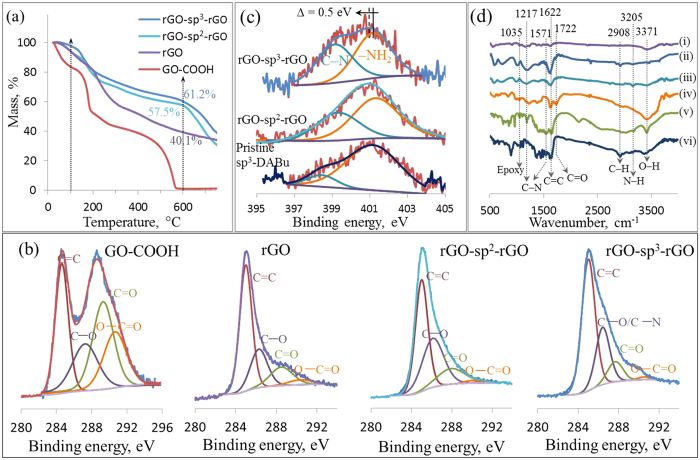
TGA curves (**a**) core level of C1s XPS spectra (**b**) of GO–COOH, rGO, rGO-sp^2^-rGO and rGO-sp^3^-rGO, the high resolution N1s spectra (**c**) of pristine sp^3^-DABu, rGO-sp^2^-rGO and rGO-sp^3^-rGO, and FTIR of rGO (i), rGO-sp^3^-rGO (ii), rGO-sp^2^-rGO (iii), GO (iv), sp^2^-DABe (v), sp^3^-DABu (vi) (**d**).

The high-resolution C1s XPS spectra (Fig. 2b) of all samples represented the defective sp^3^-C atoms and basal-plane sp^2^-C atoms of rGO^11^. GO–COOH C1s spectrum (Fig. 2b) showed four absorbance peaks for oxygenated sp^3^-C at 286.6, 287.8, and 289.4 eV, which were attributed to C–O, C=O, and O–C=O, respectively, including distinct oxygen-free sp^2^-C (C=C) at 285.0 eV^1^,^43^. rGO, rGO-sp^2^-rGO, and rGO-sp^3^-rGO samples showed stronger suppression for the oxygen-containing components of their C1s XPS spectra than GO–COOH and GO samples. These results indicated an efficient reduction of the oxygen-containing functional groups by NaBH_4_. The sp^2^/sp^3^ ratios of rGO-sp^3^-rGO and rGO-sp^2^-rGO decreased to 2.4 and 3.4, respectively, compared with those of nonfunctionalized rGO (3.7). This result indicated that the percentage of sp^3^-C in rGO-sp^3^-rGO increased relative to that in rGO-sp^2^-rGO and rGO because of the presence of sp^3^-DABu (Figure S2a). More importantly, O–C=O reduced tremendously to 1.76% and 1.36% in the rGO-sp^2^-rGO and rGO-sp^3^-rGO samples, respectively, relative to rGO (4.2) probably because of the functional molecules grafting earlier through covalent amidation. By contrast, the NaBH_4_ treatment was common in all samples. The C–O peak of rGO-sp^2^-rGO and rGO-sp^3^-rGO was significantly stronger than that of rGO probably because of the influence of C–N on the C–O bond in both samples^8^,^9^. This result also confirmed the amidation between GO–COOH and N terminals of sp^3^-DABu or sp^2^-DABe.

Figure 2c shows the N1s XPS spectra of rGO-sp^2^-rGO, rGO-sp^3^-rGO, and pure sp^3^-DABu with two absorbance peaks. In pure sp^3^-DABu, absorbance peaks at 401.05 and 398.4 eV were attributed to the amine (–NH_2_) and pyridinic-like N (C–N–C) bonds, respectively^44^,^45^,^46^,^47^. In the rGO-sp^2^-rGO (N content 7.85 wt%) and rGO-sp^3^-rGO (N content 7.87 wt%) samples, the peaks were located at 401.05 and 399.2 eV, respectively. After amidation, the pyridinic-like N peak intensity increased substantially, again confirming the amidation between GO–COOH and N terminals of sp^3^-DABu or sp^2^-DABe. The overall N1s peak for rGO-sp^2^-rGO and rGO-sp^3^-rGO negatively shifted to a lower binding energy at 401 eV (Δ*E* = 0.5 eV) than that for pure sp^3^-DABu, thereby indicating the electron transfer from the C atom of the graphitic plane to the N atom through sp^3^-DABu or sp^2^-DABe^9^,^46^. Thus, sp^3^-DABu or sp^2^-DABe acted as the junction that caused the partial electron transfer from electron-rich rGO sheets. However, it is reported that the pyridinic-like N play a critical role in determining the ORR activity among all kinds of N configurations in N-containing rGO^15^,^45^. The highest content of pyridinic-like N was found in the rGO-sp^3^-rGO (42.7%) compared to rGO-sp^2^-rGO (33.9%) (Figure S2b). Therefore, it is expected that the rGO-sp^3^-rGO sample could have the better electrocatalytic ORR activity. The conversion treatment of GO for preparing GO–COOH was significantly enhanced the amidation reaction as shown in Figure S3.

Figure 2d shows Fourier transform infrared (FTIR) spectra of all above samples including pristine sp^3^-DABu and sp^2^-DABe. Several peaks at 3371, 1722, 1622, 1035 cm^−1^ were assigned to the O–H, C=O, C=C and epoxy groups in GO spectrum. Unlikely, all above peaks were reduced or disappeared in other rGO containing samples except 1622 cm^−1^ due to reduction reaction^14^. For rGO-sp^2^-rGO and rGO-sp^3^-rGO, the peaks at 1217 and 1571 cm^−1^ were attributed to the C–N group^47^ which was also appeared in sp^3^-DABu or sp^2^-DABe samples which confirming the C–N bond formation in both samples. Moreover, the peak at 3205 cm^−1^ can be assigned to the N–H bond in rGO-sp^2^-rGO and rGO-sp^3^-rGO samples^48^. The peak at 2908 cm^−1^ for aliphatic C–H can be appeared on rGO-sp^3^-rGO sample due to aliphatic sp^3^-DABu attachment in between rGO layers. This result confirms the enhanced C–N bond formation in rGO-sp^2^-rGO and rGO-sp^3^-rGO samples and presence of the aromatic (sp^2^-DABe) and aliphatic (sp^3^-DABu) molecules, respectively.

**Figure 3:**
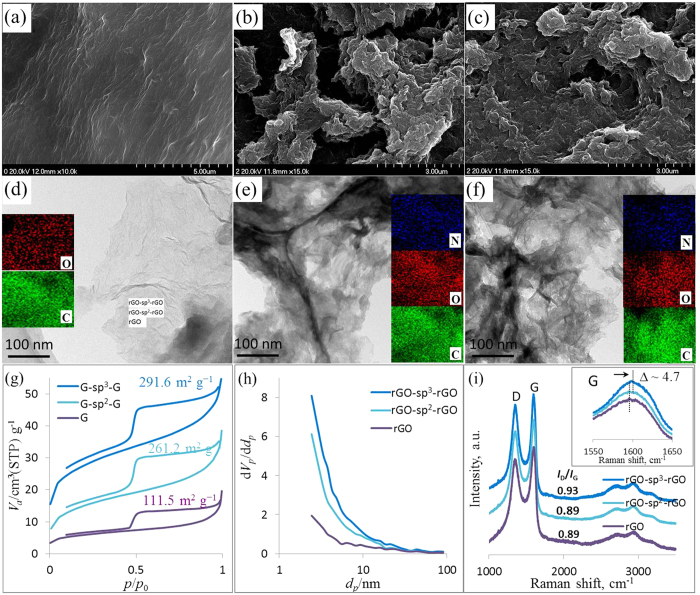
SEM (up) and TEM (down) images of rGO (**a** and **d**), rGO-sp^3^-rGO (**b** and **e**) and rGO-sp^2^-rGO (**c** and **f**), the BET surface area calculated from nitrogen adsorption–desorption isotherms (**g**), the corresponding pore-size distribution (**h**), the Raman spectroscopy (**i**); inset: the elemental mapping of C, O and N; and the magnified Raman spectra at G band region (**c**).

Figure 3 illustrates the surface morphology of rGO, rGO-sp^2^-rGO, and rGO-sp^3^-rGO, which all consisted of film-like graphene planes. The SEM image of rGO (Fig. 3a) shows a slightly wrinkled, sheet-like structure within the synthesized rGO films. The SEM image of rGO-sp^3^-rGO (Fig. 3b) showed randomly distributed, considerably crumpled structures within the synthesized rGO-sp^3^-rGO. These structures were most likely caused by the attachment of the N atom to the rGO plane as a result of covalent functionalization via amidation^31^. The substantially crumpled rGO-sp^3^-rGO films were interconnected with one another through sp^3^-DABu to form a porous 3D structure, thereby effectively preventing the aggregation of 2D graphitic sheets. These structures contributed to the enhancement of the electromechanical properties of the film and were beneficial to mechanical/thermal stability^37^,^38^,^49^. Similar morphology was observed in rGO-sp^2^-rGO only with a less pronounced porous structure (Fig. 3c).

The morphology was further investigated with TEM. Figure 3d shows the silky, wave-like morphology of a single-layer graphene film, which was similar to its SEM image. Porous rGO-sp^3^-rGO and rGO-sp^2^-rGO (Fig. 2e and f) showed a crumpled, veil-like morphology with a large amount of wrinkles and multi-layered structure similar to those of other 3D graphene^50^. Two or more sheets were found stacked unlike in Fig. 3d. Elemental mapping of C and O atoms (figure inset) for rGO showed that the density of O is considerably lower than that of the C atom because of the reduction treatment. Elemental mapping of C, O, and N elements for functionalized samples are presented in the corresponding Figure insets. A homogeneous distribution of N atoms was identified in the entire rGO-sp^3^-rGO and rGO-sp^2^-rGO samples, thereby indicating that sp^3^-DABu or sp^2^-DABa was incorporated into the whole rGO sheets through covalent amidation reaction.

Figure 3g shows the results of the N_2_ absorption/desorption analysis performed to characterize further the porous structure of the synthesized rGO, rGO-sp^2^-rGO, and rGO-sp^3^-rGO. Specific surface area (SSA) is an important parameter in understanding the electrochemical properties of porous 3D graphene materials^51^. The SSA of rGO, rGO-sp^2^-rGO, and rGO-sp^3^-rGO was determined as 111.2, 261.2, and 289.6 m^2^ g^−1^, respectively, by Brunauer-Emmett-Teller (BET) method. The SSA of rGO-sp^3^-rGO was 2.6 times higher than nonfunctionalized rGO. Pore size distributions of rGO, rGO-sp^2^-rGO, and rGO-sp^3^-rGO were also derived from the adsorption branches of isotherms with the Barrett–Joyner–Halenda model (Fig. 3h). All samples showed a prominent pore size distribution in the range of 2–3 nm, which could be ascribed to the nanopores in the graphitic basal plane of rGO. However, compared with rGO, rGO-sp^2^-rGO and rGO-sp^3^-rGO both showed prominent pore size distributions of up to 60 nm, thereby confirming the hierarchical porous structure of both catalysts. This finding corresponded with the morphological analysis.

On the basis of the above characterization, we were unable to find any significant difference between rGO-sp^2^-rGO and rGO-sp^3^-rGO except for the high sp^3^-C content and SSA. Therefore, we further investigated these differences by Raman spectroscopy, which is an important tool in the investigation of surface electronic properties of graphene-based materials^11^,^52^. Raman spectra usually have two major bands: the D band (at approximately 1350 cm^−1^), which is a breathing mode of ƙ-point phonons of the *A*_1g_ symmetry, and the G band (at approximately 1590 cm^−1^), which is assigned to the *E*_2g_ phonon of the sp^2^-C atoms. A prominent D band in the Raman spectrum is an indicator of a graphitic plane disorder originating from the sp^3^-C atoms and/or heteroatoms associated in the sp^2^-C network in plane^11^,^53^. Curves in Fig. 3i show that the *I*_D_/*I*_G_ increased from 0.89 (for rGO) to 0.93 (for rGO-sp^3^-rGO) because of the covalent attachment of sp^3^-DABu, whereas *I*_D_/*I*_G_ remained constant for rGO-sp^2^-rGO (0.89), which was covalently functionalized by sp^2^-DABa. This observation indicated that the increase in defects in the sp^2^-C network in rGO was probably caused by the sp^3^-C attachment in sp^3^-DABu. The N atom in both sp^3^-DABu and sp^2^-DABa was covalently grafted as a heteroatom on the graphitic plane. This result indicated that the sp^3^-DABu addition through covalent amidation significantly increased the defects by increasing sp^3^-C and N heteroatoms. Unlike the G band of rGO-sp^3^-rGO was up-shifted to 1598.9 cm^−1^, compared to the G band of rGO-sp^2^-rGO (1594.9 cm^−1^) and rGO (1594.1 cm^−1^). This result is an important indication of the p-type functionalization of graphene and the similar blue-shift have been reported for graphene functionalized with other electron-accepting molecules^8^,^9^,^54^. This blue-shift was caused by a charge transfer to the N atom from adjacent C atoms of graphitic plane. Therefore, Raman analysis suggested a better charge transfer to the N atom in rGO-sp^3^-rGO than that in the rGO-sp^2^-rGO and nonfunctionalized rGO due to the presence of electron-withdrawing pyridinic-type N^14^ which was confirmed by XPS analysis in Fig. 2c.

Important for electrical conductivity and electrocatalytic features, the interfacial properties of the modified electrode were first analyzed by electrochemical impedance spectroscopy (EIS) measurement. The EIS experiment was conducted in an Ar-saturated 5 mM [Fe(CN)_6_]^3−/4−^ redox probe containing 0.1 M KCl in the frequency of 10^5^–10^−2^ Hz (Figure S5). Electron transfer characteristics were interpreted by Randle’s circuit, which consists of electrolyte resistance (R_s_), electron transfer resistance (R_ct_), double-layer capacitance (C_dl_), and Warburg impedance (W) (Figure S5 inset). The Nyquist plot of rGO-modified glassy carbon electrode (rGO/GCE) displayed a well-defined, enlarged semicircle with an R_ct_ of approximately 61 Ω at high frequency. Unlike that in rGO/GCE, the diameter of the semicircle in the Nyquist plot of rGO-sp^2^-rGO/GCE and rGO-sp^3^-rGO/GCE decreased and featured an R_ct_ of 56 Ω and 50.5 Ω, respectively. This result indicated that rGO-sp^3^-rGO is a better conducting material and could act as a conductive plane to promote the electron transfer. According to the Raman and EIS analyses, a significant difference could be found in the charge transfer. Presumably, the interlayer charge transfer in the 3D rGO-sp^2^-rGO material could be initially hindered by the π-electron cloud in sp^2^-DABe. Conversely, the easy interlayer charge transfer in 3D rGO-sp^3^-rGO could be due to the absence of π-electron cloud in sp^3^-DABu.

**Figure 4:**
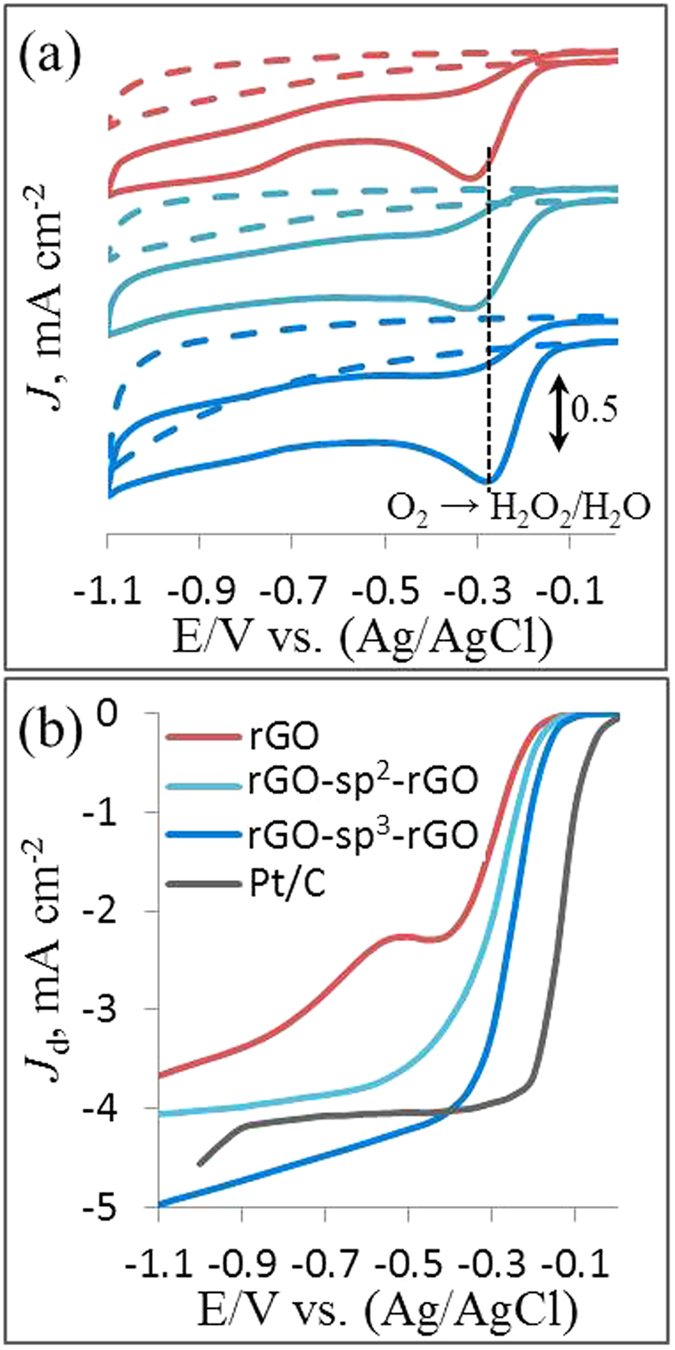
The CVs of ORR on the rGO, rGO-sp^2^-rGO, and rGO-sp^3^-rGO at 50 mV s^−1^ scan rate (**a**), and the LSV of ORR at 2500 rotation speed on rGO, rGO-sp^2^-rGO, rGO-sp^3^-rGO and Pt/C (**b**) at a scan rate of 10 mV s^−1^ in Ar- (dotted lines) O_2_- (solid lines) saturated 0.1 M KOH.

CVs for the different electrodes were measured in Ar- and O_2_-saturated 0.1 M KOH solutions at a constant active mass loading to investigate the electrocatalytic activities of the synthesized rGO, rGO-sp^2^-rGO, and rGO-sp^3^-rGO samples. Figure 4a shows the ORR peaks observed for all the modified electrodes in O_2_-saturated 0.1 M KOH solutions. Two well-defined cathodic reduction peaks at −0.32 V and −0.84 V were observed in the rGO produced curve. This was caused by the H_2_O_2_ production during indirect (two-step) O_2_ reduction. These two peaks were assigned to the two-electron transfer ORR followed by H_2_O_2_ reduction reaction^8^,^13^,^21^,^55^. Compared with the rGO electrode, the H_2_O_2_ reduction peak disappeared with a pronounced increase in the ORR peak at the rGO-sp^2^-rGO electrode, thereby indicating a direct ORR mechanism. However, a single and prominent peak assigned to O_2_ reduction appeared in the sp^3^-DABu functionalized rGO-sp^3^-rGO produced CV curve. Unlike that for rGO and rGO-sp^2^-rGO, the peak potential for the rGO-sp^3^-rGO electrode shifted positively to −0.28 V. After correcting the background current, the current density was measured as 0.71 mA cm^−2^ and was obviously higher than that of the rGO (0.62 mA cm^−2^) and rGO-sp^2^-rGO (0.66 mA cm^−2^) electrodes. The corresponding H_2_O_2_ reduction peak (at −0.84 V) also disappeared at the rGO-sp^3^-rGO electrode, thereby indicating a direct (single-step) four-electron transfer ORR occurred in both N atoms containing rGO-sp^2^-rGO and rGO-sp^3^-rGO electrodes, in which H_2_O is the main product^43^. These results clearly indicated that not only the ORR catalytic activity of rGO-sp^2^-rGO and rGO-sp^3^-rGO was greater than that of rGO but also the favorable four-electron involved direct ORR mechanism could be attributed to covalent amidation.

Linear sweep voltammetry (LSV) measurements were performed on a rotating-disk electrode (RDE) in O_2_-saturated 0.1 M KOH solution at a constant rotation speed of 2500 rpm to evaluate further ORR electrocatalytic activity of rGO, rGO-sp^2^-rGO, rGO-sp^3^-rGO, and Pt/C in Fig. 4b. Similar to other nonfunctionalized rGO^9^, the rGO electrode showed a two-step, two-electron involved ORR pathway with an onset potential (*E*_onset_) of −0.15 V. Unlike the rGO electrode, rGO-sp^2^-rGO exhibited a single-step, four-electron involved ORR pathway with a positively shifted *E*_onset_ at −0.14 V with high limiting diffusion current density (*J*_L_, mA cm^−2^). Conversely, the sp^3^-DABu-functionalized rGO-sp^3^-rGO exhibited a single-step, four-electron involved ORR pathway with a positively shifted *E*_onset_ at −0.1 V and with 1.4 times higher *J*_L_ than the nonfunctionalized rGO. The *E*_onset_ was even better than those of other metal-free electrodes^9^,^11^,^16^,^24^. The steady-state *J*_L_ catalyzed by the rGO-sp^3^-rGO electrode was higher than that of the commercially available Pt/C electrode at large potential range (Fig. 4b). Undoubtedly, the improved SSA, hierarchical porous structure, and sp^3^-amide linkage between rGO layers which facilitate interlayer charge transfer in rGO-sp^3^-rGO play key roles in enhancing its ORR activity.

**Figure 5:**
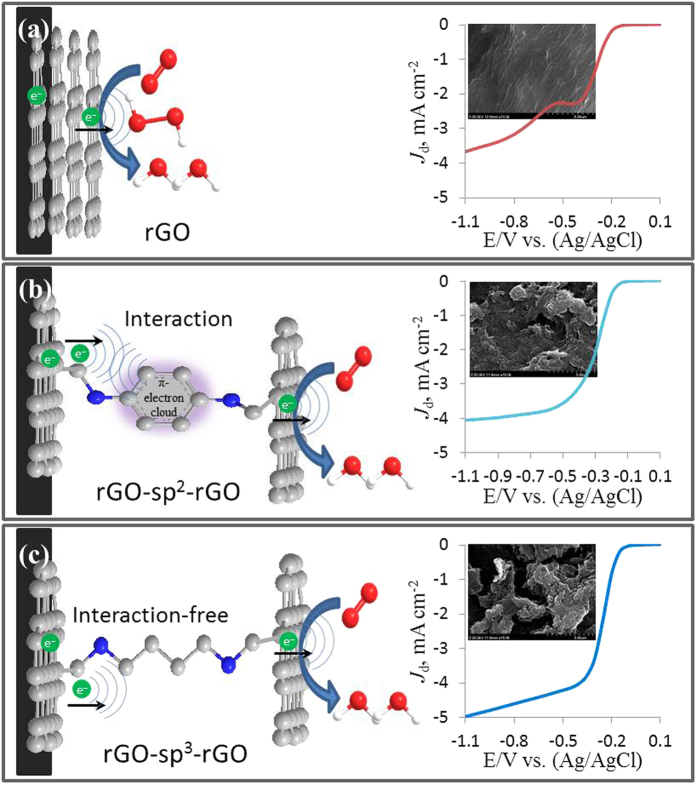
The hypothetical ORR mechanism at all catalysts.

Based on the above experimental results, the hypothetical ORR mechanism could be explained as shown in the Fig. 5. The better ORR was observed in N-rich 3D graphene catalysts (rGO-sp^2^-rGO and rGO-sp^3^-rGO) over N-free rGO catalyst (Fig. 5a) because of the covalent attachment of pyridinic-type N atom, which created a net positive charge on the adjacent C atoms to facilitate ORR. However, in consideration of rGO-sp^2^-rGO, the rGO layers were linked by the sp^2^-DABe, which has the π-electron cloud in between the rGO layers. Thus, the interlayer charge transfer could be initially hindered by the π-electron cloud when it passes through to the N atom (Fig. 5b). A small resistance by the π-electron cloud occurs in rGO-sp^2^-rGO catalyst which hinders the superior ORR. Conversely, the rGO layers were covalently linked in rGO-sp^3^-rGO by the sp^3^-DABu, which had no π-electron in between the rGO layers (Fig. 5c). Therefore, the π-electron interaction-free interlayer charge transfer could facilitate ORR.

**Figure 6:**
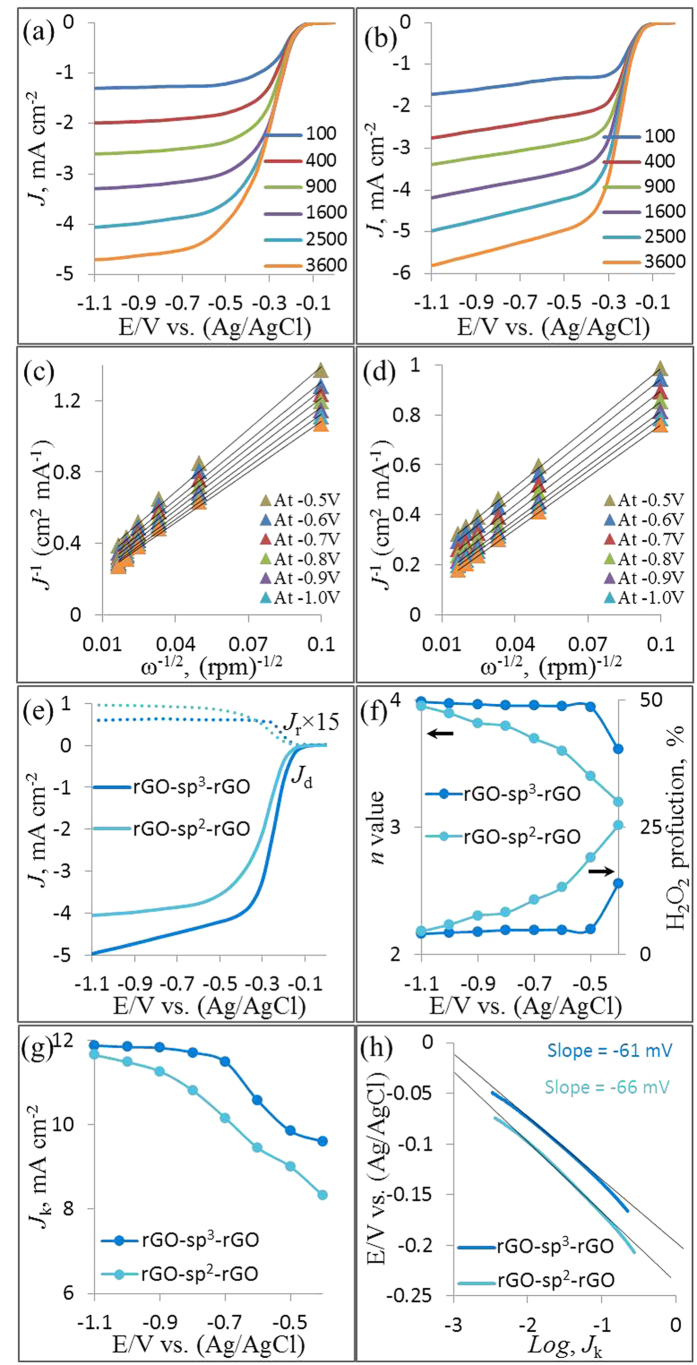
The LSV for ORR at various rotation speeds on rGO-sp^2^-rGO (**a**) and rGO-sp^3^-rGO (**b**) in O_2_-saturated 0.1 M KOH at a scan rate of 10 mV s^−1^, Koutecky-Levich plots for rGO-sp^2^-rGO (**c**) and rGO-sp^3^-rGO (**d**) at different electrode potentials, the RRDE voltammograms for ORR in O_2_-saturated 0.1 M KOH at a scan rate of 10 mV s^−1^, the ring electrode potential was constant at 1.2 V (**e**), the dependence of the transferred electron number with corresponding H_2_O_2_ formation (**f**), the kinetic current density (**g**) and the Tafel plots (**h**) of rGO-sp^2^-rGO and rGO-sp^3^-rGO electrodes.

RDE voltammetry measurements were also conducted for rGO-sp^2^-rGO and rGO-sp^3^-rGO in O_2_-saturated 0.1 M KOH solution to gain insight into the ORR kinetics. Figure 6a and b show the LSV curves at various rotation speeds for rGO-sp^2^-rGO and rGO-sp^3^-rGO electrodes, respectively. The *J*_L_ increased with the increasing rotation rate for both modified electrodes. At any constant rotation rate, ORR *J*_L_ at the rGO-sp^3^-rGO electrode was consistently higher than that at the rGO-sp^2^-rGO electrode. Direct ORR for both rGO-sp^2^-rGO and rGO-sp^3^-rGO was observed in every LSV curve. The kinetic current region (in potential range) was much smaller at the rGO-sp^3^-rGO electrode than at the rGO-sp^2^-rGO electrode, thus indicating that ORR was faster and more quickly gained steady state *J*_L_ at the rGO-sp^3^-rGO electrode. The LSV curve-derived Koutecky-Levich (K-L) plots followed a better linear relationship for the rGO-sp^3^-rGO electrode at various potentials than for the rGO-sp^2^-rGO-modified electrode (Fig. 6c and d). This observation indicated that the corresponding transferred electron numbers were similar during ORR and could represent first-order kinetics with respect to O_2_^56^. The transferred electron numbers (*n*) per O_2_ involved in ORR at both rGO-sp^2^-rGO and rGO-sp^3^-rGO electrodes were determined by the K-L equation, which is presented below^35^,^57^,^58^,^59^. The *n* values at the rGO-sp^3^-rGO electrode in 0.1 M KOH medium were estimated *ca.* 3.85–4 at the cited potential range (−0.4 to −1.1 V), and *ca.* 3.6–3.95 for rGO-sp^2^-rGO at the same potential range.


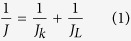



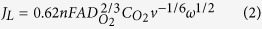


where *J*, and *J*_k_ are the measured, and kinetic current densities (mA cm^−2^), respectively; *A* is the surface area of GCE (0.196 cm^2^); *F* and *T* are the Faraday constant (96485.3 C mol^−1^) and temperature, respectively; *Do*_2_ and *Co*_2_ are the oxygen diffusion coefficient (1.9 × 10^−5^ cm^2^ s^−1^) and the bulk concentration (1.2 mmol L^−1^), respectively, in 0.1 M KOH^8^,^9^; *v* is the kinetic viscosity of the electrolyte (1 × 10^−2^ cm^2^ s^−1^); and *ω* is the rotation rate of the electrode (rpm).


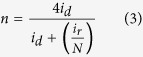



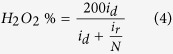



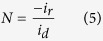


where *N* is the collection efficiency of the rotation ring–disk electrode (RRDE) (0.37), and *i*_d_ and *i*_r_ are the disk and ring electrode currents, respectively.

ORR kinetics at the rGO-sp^2^-rGO and rGO-sp^3^-rGO electrodes was further evaluated with the RRDE. Figure 6e shows the disk (down) and ring (up) current densities for both electrodes. The disk curves followed by the ring curves and produced a mirror image. Ring currents were measured at a constant set potential of 1.2 V to estimate the amount of generated H_2_O_2_^60^,^61^. Consistent results were obtained for the RRDE measurements, but however, the H_2_O_2_ amount generated at the rGO-sp^3^-rGO electrode was significantly less than that at the rGO-sp^2^-rGO electrode, thereby indicating that rGO-sp^3^-rGO is an efficient ORR electrocatalyst. Further *n* values and H_2_O_2_ yields were calculated from the given equations (3 and 4) based on RRDE data^48^,^62^,^63^. Figure 6f shows that the *n* value increased as the negative potential increased. The *n* value for ORR at the rGO-sp^3^-rGO electrode (3.95 to 3.98) was consistently higher than that at the rGO-sp^2^-rGO (3.4 to 3.95) over the tested potential range (−0.5 to −1.1 V). The nearly constant *n* value for long potential range is rarely observed at metal-free electrodes (Table S1). These findings are consistent with those obtained from the K–L plots based on the RDE measurements. The corresponding H_2_O_2_ yield for rGO-sp^3^-rGO was 5–4%, and a high H_2_O_2_ yield was calculated at rGO-sp^2^-rGO (18–4.5%) over the examined potential range. This result is also consistent with the relatively higher calculated kinetic current density (calculated from equation 1) for ORR at the rGO-sp^3^-rGO electrode with respect to the rGO-sp^2^-rGO electrode (Fig. 6g). Based on the *n* value, kinetic current density, and H_2_O_2_ formation, a dominant and direct ORR process involving four electrons clearly occurred at the rGO-sp^3^-rGO catalyst.

Finally, the estimated *J*_k_ values were plotted against the potential to analyze the Tafel behavior of the tested catalysts (Fig. 6h). Tafel slopes were estimated as 61 and 66 mV dec^−1^ for rGO-sp^3^-rGO and rGO-sp^2^-rGO, respectively. The 60 mV dec^−1^ Tafel slope was frequently exhibited by state-of-the-art Pt/C for ORR at a low overpotential region^14^, and it could be attributed to the adsorbed O_2_ with the fast initial electron transfer step followed by the rate-determining step^64^,^65^. The lower Tafel slope of rGO-sp^3^-rGO than that of rGO-sp^2^-rGO further confirmed the superior and faster electrocatalytic ORR performance of rGO-sp^3^-rGO^66^.

**Figure 7:**
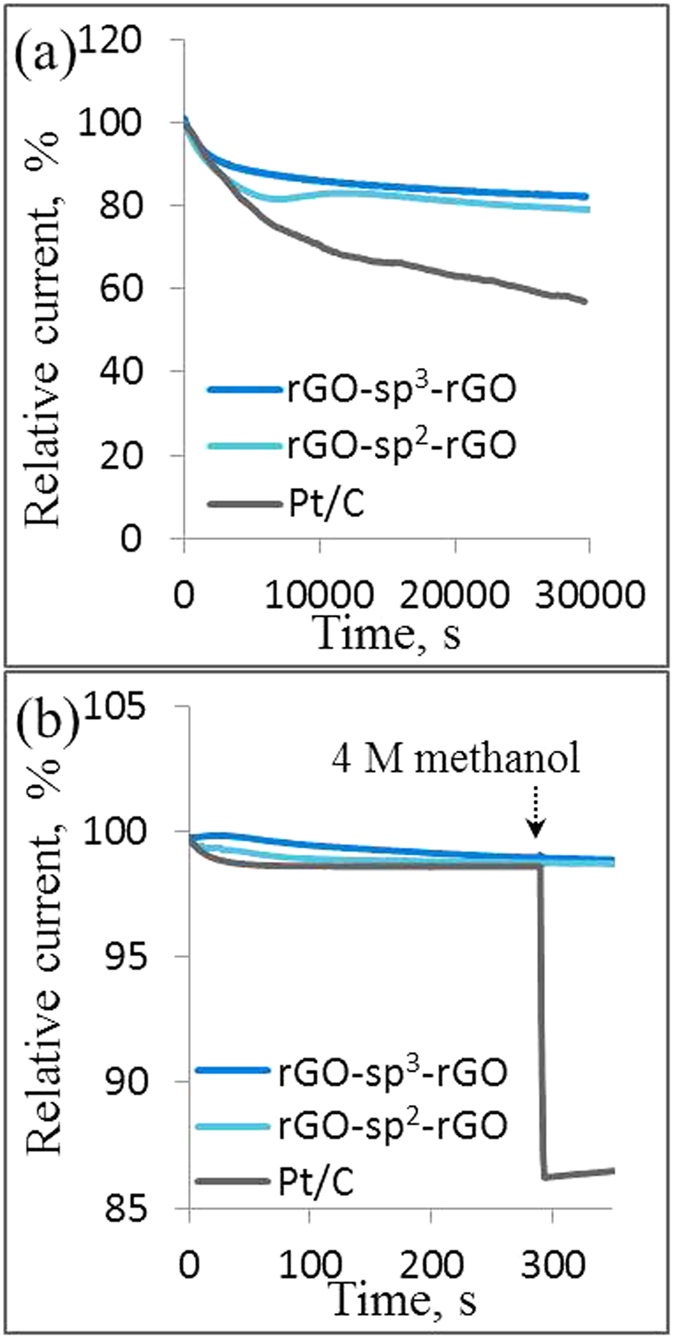
The long term CA responses for ORR at an applied potential of −0.3 V and at 1600 rpm (**a**) and the methanol selectivity test upon 4 M methanol addition (**b**) on rGO-sp^2^-rGO, rGO-sp^3^-rGO and Pt/C electrodes.

The long-term stability of rGO-sp^2^-rGO, rGO-sp^3^-rGO, and Pt/C catalysts was also evaluated because of their importance in FCs application. The catalysts were held at −0.3 V in an O_2_-saturated 0.1 M KOH solution for approximately 8 h at 1600 rpm (Fig. 7a). Figure 7a displays the current versus time CA response for rGO-sp^2^-rGO and rGO-sp^3^-rGO. rGO-sp^2^-rGO and rGO-sp^3^-rGO showed a high relative current of 83% and 88.5%, respectively, which persisted after 30,000s. By contrast, the Pt/C electrode exhibited a gradual decrease with a current loss of approximately 57% after the same period. The stability of three different rGO-sp^3^-rGO catalysts prepared with three different w/w ratios of GO–COOH and sp^3^-DABu was also compared (Figure S6). The CA technique was also used to examine the application potentiality in direct alcohol FCs by investigating methanol poisoning effects. The fuel selectivity of rGO-sp^2^-rGO, rGO-sp^3^-rGO, and Pt/C was measured in 4 M methanol containing O_2_-saturated 0.1 M KOH solution. No significant response was observed in the ORR current of rGO-sp^2^-rGO and rGO-sp^3^-rGO after the addition of 4 M methanol (Fig. 7b). By contrast, a substantial decrease (87%) in the activity of Pt/C was detected in the presence of 4 M methanol under the same experimental conditions. These results confirmed that rGO-sp^2^-rGO and rGO-sp^3^-rGO possessed high stability and selectivity for ORR with remarkable tolerance to methanol poisoning, thereby further indicating the potentiality of rGO-sp^3^-rGO catalysts to replace noble metal catalysts.

## Conclusion

Through covalent amidation reaction, we successfully prepared a metal-free ORR catalyst based on pyridinic-like N-rich functionalized rGO (rGO-sp^2^-rGO and rGO-sp^3^-rGO) with excellent ORR performance, high SSA, and conductivity over nonfunctionalized rGO. Especially, amide-functionalization and sp^3^-C-rich rGO-sp^3^-rGO could act as an efficient metal-free electrocatalyst because of the π-electron interaction-free interlayer charge transfer which facilitating the ORR in the hierarchical porous 3D plane. In addition to its good ORR electrocatalytic activity, our synthesized metal-free ORR catalyst also has higher stability and excellent methanol tolerance ability, thus making it a promising alternative to metal catalysts. These findings can encourage researchers to develop more cost-effective carbon-based metal-free catalysts for various applications.

## Methods

GO was prepared from graphite powder (Alderich, 325 mesh, 99.999%) using the modified Hummer’s method^28^,^67^,^68^. Figure 1 shows the GO–COOH preparation with a modified procedure^5^. Briefly, 50 mg GO and 25 mL of water were loaded into a 100 mL round-bottom flask. The resultant yellow-brown homogeneous solution was subjected to ultrasonic agitation until it became clear with no visible large particulates. Afterwards, 2.5 g of chloroacetic acid (ClCH_2_COOH, Aldrich, Korea) and 3.2 g of NaOH were added to the aforementioned solution. The solution was maintained under stirring for 3 h at room temperature (RT, approximately 25 °C). NaOH was neutralized by the addition of 2.7 mL of HCl to the solution. The solution was filtered and washed with water four times. It was subjected to 30 min of sonication in 10 ml phosphate-buffered saline (PBS, at pH 5.5) containing 40 mg 1-(3-dimethylaminopropyl)-3-ethylcarbodiimide (EDC, Aldrich, Korea) to produce a homogeneous suspension. Then, 40 mg of GO–COOH and 150 mg N-hydroxysuccinimide (NHS, Aldrich, Korea) were added. The suspension was maintained 3 h of sonication after the addition of 80 mg sp^3^-DABu. Finally, 80 mg NaBH_4_ was added to the suspension, which was refluxed at 55 °C overnight. The obtained rGO-sp^3^-rGO was filtered, washed several times with distilled water, and dried under vacuum at 40 °C for 12 h. For comparison, rGO-sp^2^-rGO was prepared with sp^2^-hybridized 1,4-diaminobenzene (sp^2^-DABe) instead of sp^3^-DABu. The rGO was prepared without addition of any functional molecules.

Transmission electron microscopy (TEM) images and energy-dispersive X-ray spectroscopy (EDX) were obtained with the TECNAI model FI-20 (FEI, Netherlands). Scanning electron microscope (SEM) images were obtained with a JSM-7500F field emission scanning electron microanalyzer (JEOL). Raman spectra were obtained with the LabRam HR800 UV Raman microscope (Horiba Jobin-Yvon, France) with an excitation of 514 nm Ar^+^ laser. BET surface area and pore size distribution were obtained through the Barrett-Joyner-Halenda method by nitrogen isotherm adsorption and desorption (BelsorpII mini, BEL Japan Inc.). X-ray photoelectron spectroscopic (XPS) images were obtained by MultiLab 2000 with a 14.9 keV Al K X-ray source. Curve fitting was conducted with the XPSPEAK41 system software. Thermogravimetric analysis (TGA) was performed in a nitrogen atmosphere with a TGA-50 Thermogravimetric Analyzer (Shimadzu, Japan) at a heating rate of 20 °C/min. FTIR measurements were performed on an FTIR spectroscope (PerkinElmer).

rGO-sp^3^-rGO suspension (1 mg mL^−1^) was prepared in water through ultrasonication. A 10 μL portion of rGO-sp^3^-rGO ink was dropped onto the prepolished GCE. rGO-sp^2^-rGO- and rGO-coated GCEs were prepared following the same protocol. The commercially available Pt/C (Johnson Matthey 20 wt% on Vulcan XC-72) suspension was prepared by dispersing 1 mg mL^−1^ of Pt/C in ethanol containing 5 μL of 5% Nafion solution (in alcohol). Afterwards, 28.3 μg cm^−2^ Pt was loaded onto the GCE. All electrochemical measurements, including cyclic voltammetry (CV) and chronoamperometry (CA), were obtained with a three-electrode potentiostat (CHI 700C electrochemical workstation [U.S.A.]). The auxiliary and reference electrodes were a Pt wire and Ag/AgCl electrodes, respectively. EIS was performed with a Versa State 3 manufactured by Metek, USA). All electrochemical experiments were performed in high-purity argon (Ar) or O_2_-saturated 0.1 M KOH solutions (pH 13) at RT (approximately 25 °C).

## Additional Information

**How to cite this article**: Shamsuddin Ahmed, M. and Kim, Y.-B. 3D graphene preparation via covalent amide functionalization for efficient metal-free electrocatalysis in oxygen reduction. *Sci. Rep.* **7**, 43279; doi: 10.1038/srep43279 (2017).

**Publisher's note:** Springer Nature remains neutral with regard to jurisdictional claims in published maps and institutional affiliations.

## Supplementary Material

Supporting Information
