# Supplementary material for: 3D graphene preparation via covalent amide functionalization for efficient metal-free electrocatalysis in oxygen reduction
Source: Sci Rep. 2017 Feb 27;7:43279. doi: 10.1038/srep43279 (PMC5327434; doi:10.1038/srep43279)
Supplement: Supporting Information [file srep43279-s1.pdf]

Supporting Information of

3D graphene preparation via covalent amide functionalization for efficient metal-free electrocatalysis in oxygen reduction

Mohammad Shamsuddin Ahmed, Young-Bae Kim*

Department of Mechanical Engineering, Chonnam National University, Gwangju, Republic of Korea. Tel.: +82 62 5301677, *E-mail: ybkim@chonnam.ac.kr

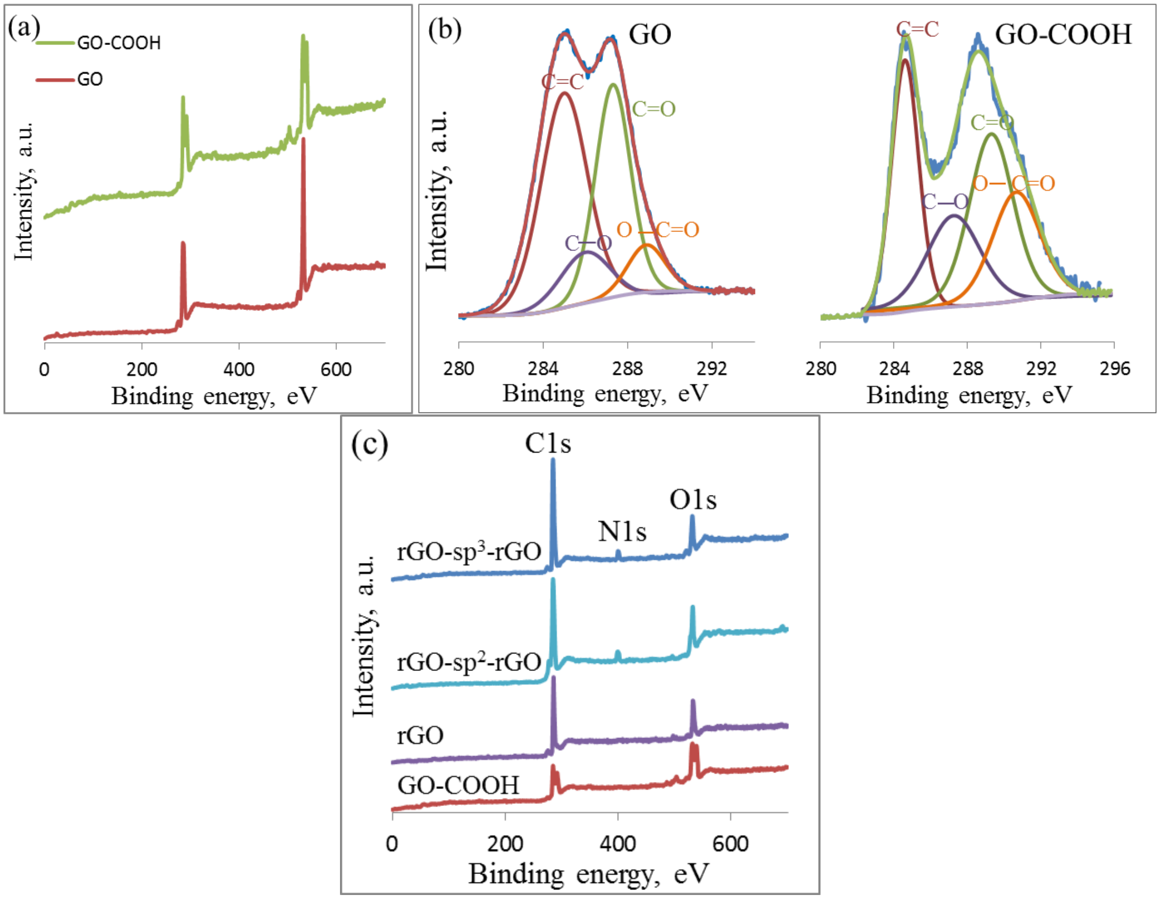

Figure S1: XPS survey spectra (a), core level of C1s spectra (b) of GO and GO-COOH and XPS survey spectra of GO–COOH, rGO, rGO-sp^2-rGO and rGO-sp^3-rGO (c).

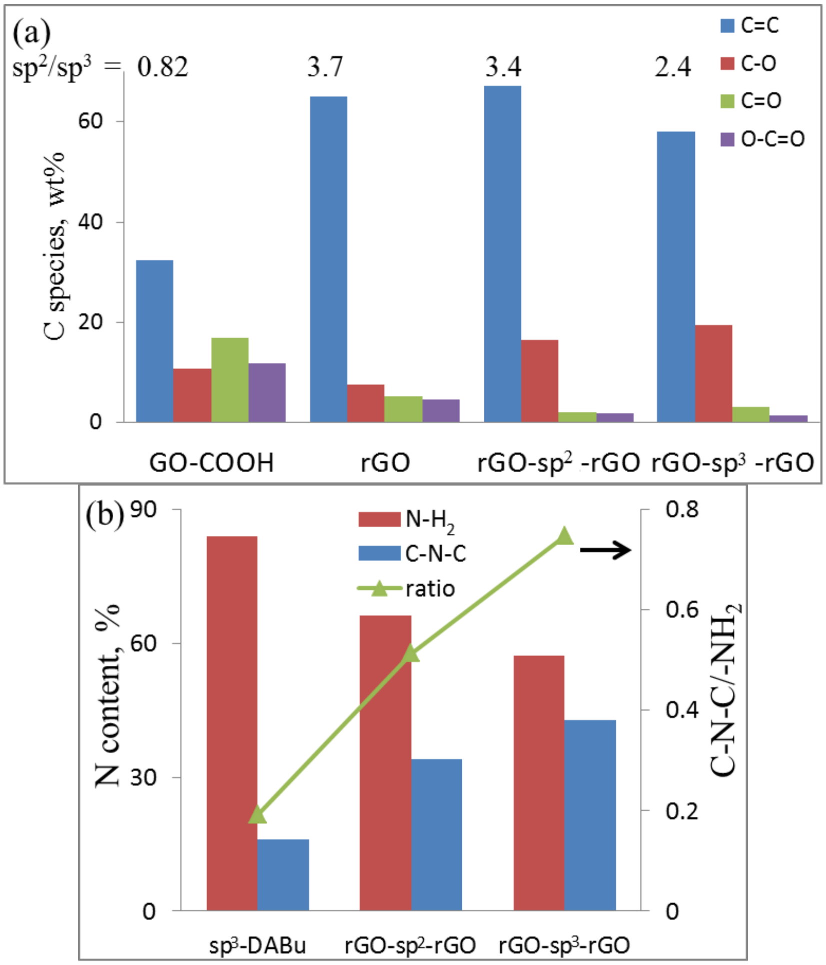

Figure S2: The C-species content calculated from the wt% of C for all samples (a) N-species content calculated from the wt% of N for sp^3-DABu, rGO-sp^2-rGO and rGO-sp^3-rGO (b).

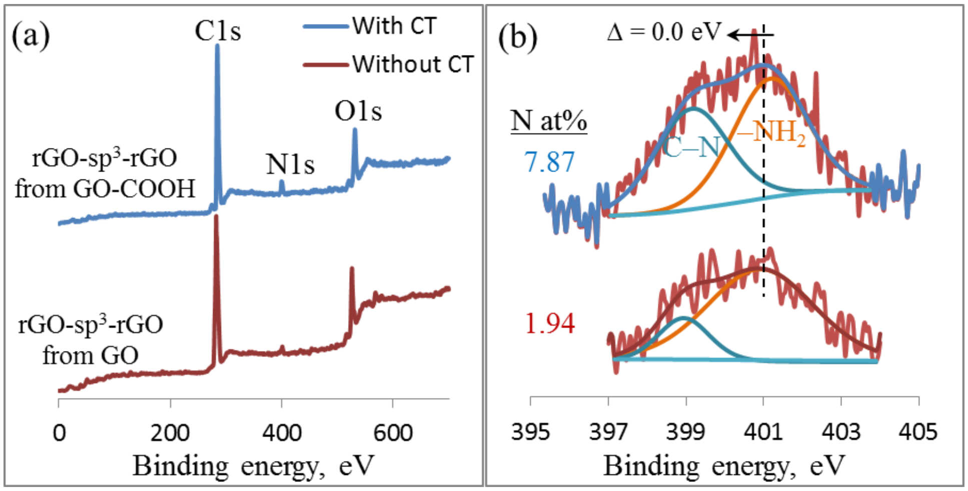

Figure S3: XPS survey spectra (a), core level of N1s spectra (b) of rGO-sp^3-rGO samples from without (GO) and with (GO-COOH) conversion treatment (CT) precursor.

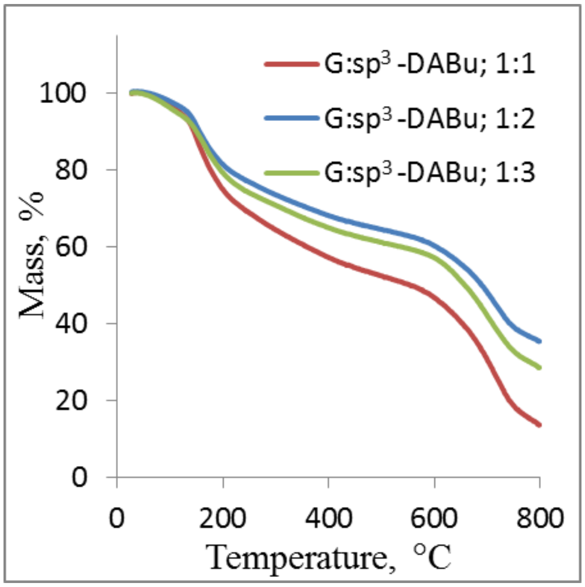

Figure S4: TGA curves of three different rGO-sp^3-rGO materials which prepared by three different w/w ratios of GO-COOH and sp^3-DABu (1:1, 1:2, and 1:3).

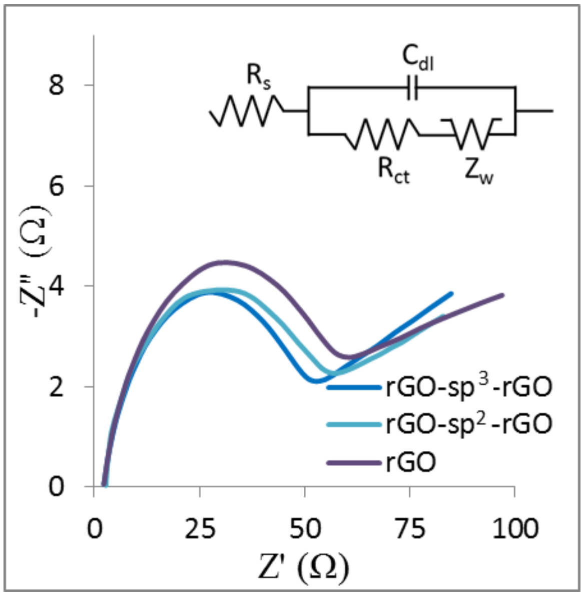

Figure S5: Nyquist plots of rGO/GCE, rGO-sp^2-rGO/GCE and rGO-sp^3-rGO/GCE.

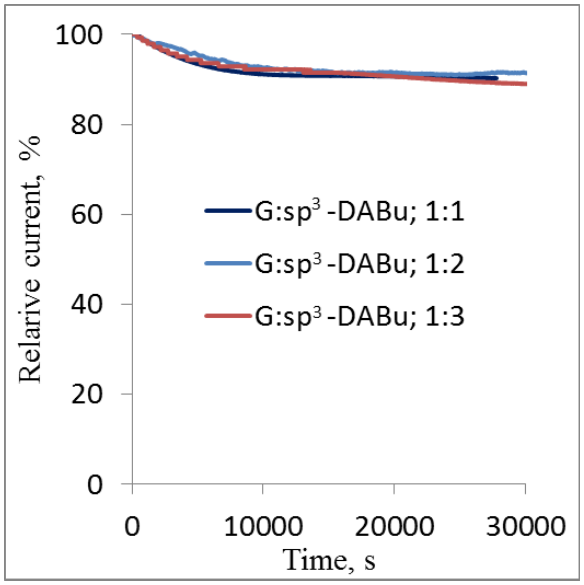

Figure S6: CA curves of three different rGO-sp^3-rGO catalysts which prepared by three different w/w ratios of GO-COOH and sp^3-DABu (1:1, 1:2, and 1:3).

Table S1: The comparison of ORR activity of the metal-free catalysts.

Catalyst	Electrolyte	E_{onset} (Ref. electrode)	*n* value	References
PDDA[a]–graphene	0.1 M KOH	–0.105 V (vs. Ag/AgCl)	3.5–4	[9]*
P–G[b]	0.1 M KOH	–0.112 V (vs. Ag/AgCl)	3.85 (0.4 V)	[11]*
N–graphene	0.1 M KOH	–0.17 V (vs. Ag/AgCl)	3.6–4	[16]
B_3CNTs[c]	1 M NaOH	–0.13 V (vs. Ag/AgCl)	2.5 (0.7 V)	[24]*
TDMAC[d]-RGO	0.1 M KOH	–0.24 V (vs. Ag/AgCl)	3–4	[32]
Acr@MWCNTs	0.1 M KOH	–0.14 V (vs. Ag/AgCl)	3.2 (–1 V)	[33]
NS-C[e]-1100	0.1 M KOH	0.05 V (vs. Ag/AgCl)	3.4–3.8	[34]*
AG[f]	0.1 M KOH	–0.04 V (vs. Ag/AgCl)	3.3–4	[41]
Py[g]-EGO	0.1 M KOH	–0.2 V (vs. Ag/AgCl)	3.74 (–0.4 V)	[42]
S-graphene[h]-1050	0.1 M KOH	–0.08 V (vs. Ag/AgCl)	3.82 (–0.3 V)	[43]
NPC[i]	0.1 M KOH	−0.04 (vs. Ag/AgCl)	3.7 (–0.6 V)	[60]
rGO-sp^3-rGO	0.1 M KOH	−0.1 V (vs. Ag/AgCl)	3.95–3.98	This work

[a]poly(diallyldimethylammonium chloride), [b]plasma-treated graphene, [c]boron-doped carbon nanotibes, [d]tridodecylmethylammonium chloride, [e]sulfur and nitrogen codoped carbon, [f]amino-functionalized graphene, [g]pyridine functionalized graphene, [h]sulfur-doped graphene, [i]nitrogen self-doped porous carbon, *converted V vs. Ag/AgCl.
